# Pediatric Solid Pseudopapillary Neoplasm With Aberrant CD31 Expression: A Potential Diagnostic Pitfall

**DOI:** 10.7759/cureus.103686

**Published:** 2026-02-15

**Authors:** Jameera Nazer, Archana G Vallonthaiel, Jyotsna Yesodharan, Naveen Viswanath

**Affiliations:** 1 Pathology, Amrita Institute of Medical Sciences, Kochi, IND; 2 Pediatric Surgery, Amrita Institute of Medical Sciences, Kochi, IND

**Keywords:** biopsy, diagnostic errors, immunohistochemistry staining, pancreatic neoplasms, solid pseudopapillary neoplasm of the pancreas

## Abstract

Solid pseudopapillary neoplasm (SPN) of the pancreas is a rare epithelial tumor with low malignant potential, predominantly affecting young women and only rarely encountered in the pediatric population. Although the prognosis is generally favorable following complete surgical excision, diagnostic challenges may arise due to unusual histomorphology or aberrant immunohistochemical expression.

We report the case of a seven-year-old female patient who presented with abdominal pain and vomiting and was found to have a large intra-abdominal mass initially suspected to be of mesenteric origin on imaging. An incisional biopsy revealed extensive necrosis and viable tumor cells with epithelioid morphology, intracytoplasmic lumina, and focal CD31 positivity, raising the possibility of a vascular neoplasm. Following complete surgical excision, histopathological examination demonstrated focal pseudopapillary architecture. Targeted immunohistochemistry showed tumor cell positivity for cytokeratin, nuclear beta-catenin, CD56, dot-like CD99, progesterone receptor (focal), and aberrant focal CD31 expression, with negativity for ERG and other vascular markers, confirming the diagnosis of SPN. The patient subsequently developed metastatic disease and was started on systemic chemotherapy.

This case highlights an unusual pediatric presentation of SPN with aberrant CD31 expression, which represents a significant diagnostic pitfall, particularly in limited or necrotic biopsy specimens. Awareness of such atypical immunophenotypic findings, along with careful morphological assessment and use of a comprehensive immunohistochemical panel, is essential to avoid misdiagnosis and ensure appropriate clinical management.

## Introduction

Solid pseudopapillary neoplasm (SPN) of the pancreas is a rare epithelial tumor, accounting for approximately 2-3% of all pancreatic neoplasms [1]. First described by Frantz in 1959, SPN is now recognized as a distinct clinicopathological entity characterized by low malignant potential and a generally favorable prognosis following complete surgical excision [2]. The tumor predominantly affects young women, most commonly in the second and third decades of life, with a marked female preponderance, suggesting a possible hormonal influence on tumor development [3].

SPN can occur at any age, including childhood; however, pediatric cases are distinctly uncommon, comprising a small subset of reported cases [4]. In children, SPN often presents with nonspecific symptoms such as abdominal pain, vomiting, or a palpable mass, and may be discovered incidentally during imaging performed for unrelated reasons [5]. Radiologically, SPN typically appears as a large, well-circumscribed mass with a pseudocapsule and varying proportions of solid and cystic components, reflecting hemorrhage and necrosis within the tumor [6]. These imaging features, while suggestive, are not pathognomonic and frequently overlap with other pancreatic or intra-abdominal neoplasms, particularly in pediatric patients.

Histologically, SPN demonstrates a characteristic combination of solid sheets of loosely cohesive tumor cells and pseudopapillary structures formed by degeneration of tumor cell clusters around delicate fibrovascular cores [7]. Additional features may include extensive necrosis, hemorrhage, foamy macrophages, cholesterol clefts, calcification, and occasional multinucleated giant cells [8]. The immunohistochemical profile of SPN is distinctive and plays a crucial role in diagnosis, with consistent nuclear accumulation of beta-catenin due to CTNNB1 mutations, along with positivity for CD56, vimentin, and progesterone receptor, and characteristic dot-like cytoplasmic expression of CD99 [9,10]. Loss of membranous E-cadherin expression is also a well-recognized finding [11].

Despite these characteristic features, SPN can pose significant diagnostic challenges, particularly when biopsy specimens are limited or tumors show extensive necrosis or degenerative changes [12]. In such settings, unusual histomorphology or aberrant immunohistochemical expression may lead to misdiagnosis as other pancreatic or mesenteric neoplasms, including neuroendocrine tumors, acinar cell carcinoma, or vascular tumors [13]. Awareness of potential immunophenotypic variability is therefore essential, as misclassification can have important implications for patient management and prognosis.

We present an exceptionally rare pediatric case of pancreatic SPN with aberrant CD31 expression, an endothelial marker not typically associated with SPN. To our knowledge, such expression has not been well documented in the literature and may represent a diagnostic pitfall, particularly in small biopsy specimens. This case underscores the importance of integrating clinical, radiological, morphological, and immunohistochemical findings to arrive at the correct diagnosis.

## Case presentation

A seven-year-old female patient was admitted to our hospital with complaints of abdominal pain and non-bilious vomiting of two days' duration. The pain was predominantly localized to the left side of the abdomen, described as a dull ache, without clear aggravating or relieving factors. There was no associated loss of appetite, weight loss, fever, abdominal distension, altered bowel habits, melena, or hematochezia. There was no significant past medical history or family history suggestive of malignancy or hereditary cancer syndromes. On physical examination, the child appeared active. Anthropometric measurements revealed a weight of 27 kg, a height of 142 cm, and a BMI of 13.4 kg/m², corresponding to the fifth percentile for age.

Pallor was noted clinically, while no icterus, lymphadenopathy, or edema was present. Abdominal examination revealed a palpable, firm, non-tender mass measuring approximately 7 x 8 cm in the left hypochondrial and lumbar region, without well-defined margins and restricted mobility. Biochemical investigations showed a slight elevation of CRP (Table 1).

**Table 1:** Blood investigations done at admission AST: aspartate aminotransferase, SGOT: serum glutamic-oxaloacetic transaminase, ALT: alanine aminotransferase, SGPT: serum glutamic-pyruvic Ttransaminase

Investigation	Value	Reference range (7 years)
Hemoglobin	11.8	11.5-15.5 g/dL
Total leukocyte count	8,900	5,000-14,500/mm³
Platelet count	2.6 × 10⁵	1.5-4.5 ×10⁵ /mm³
Erythrocyte sedimentation rate (ESR)	18	<20 mm/hr
C-reactive protein (CRP)	8	<5 mg/L
Serum bilirubin (total)	0.6	0.2-1.0 mg/dL
AST (SGOT)	32	<40 IU/L
ALT (SGPT)	28	<40 IU/L
Blood urea	22	10-40 mg/dL
Serum creatinine	0.5	0.3-0.7 mg/dL

An abdominal ultrasound (done at an outside hospital) revealed an iso-echoic lesion in the left hypochondrium, measuring 8 × 7.6 × 12 cm, extending to the left lumbar region and abutting the pancreas, raising the possibility of a mass lesion.

**Figure 1:**
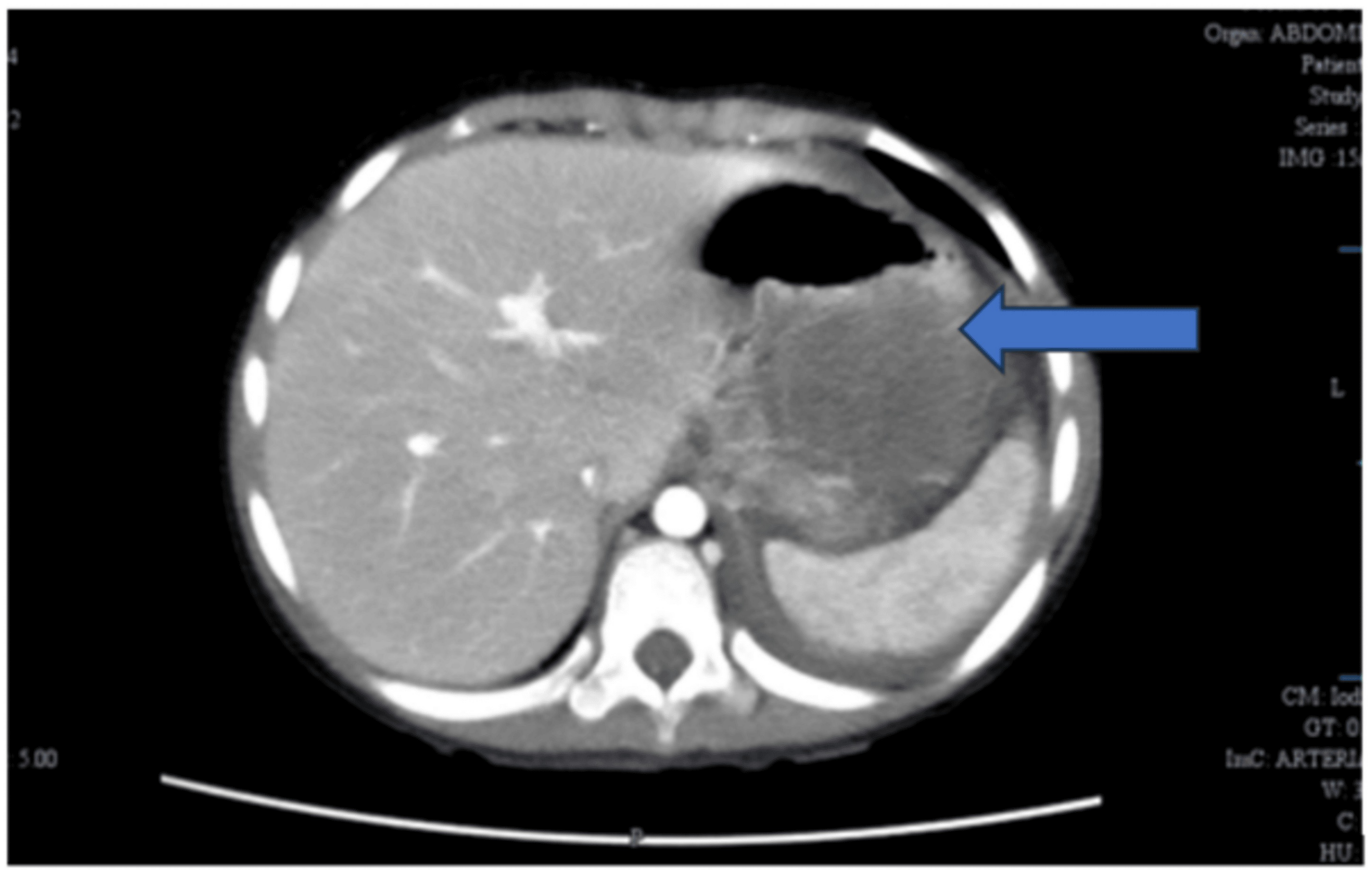
Contrast-enhanced CT scan of the abdomen (axial section) showing a well-defined mass lesion (blue arrow), adjacent to the stomach and spleen, corresponding to the clinically palpable abdominal mass CT: computed tomography

Contrast CT of the abdomen done at our center showed a large heterogenous soft tissue lesion with patchy enhancement, measuring 8.2 × 7.4 × 13.2 cm, displacing adjacent bowel loops, kidney, spleen, and tail of the pancreas with arterial supply from branches of the superior mesenteric artery, which raised the possibility of an aggressive neoplasm such as sarcoma of mesenteric/peritoneal origin (Figure 1).

The patient was admitted for surgical removal of the mass. Intraoperatively, a tumor was seen densely adherent to the transverse colon below and to the pancreas and stomach superiorly, and also to the spleen and lateral abdominal wall. In view of the dense adhesion, an incision biopsy from the mass was taken along with a side-to-side anastomosis of the transverse to the sigmoid colon.

**Figure 2:**
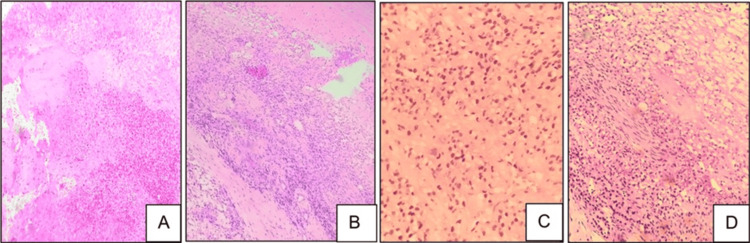
Incision biopsy showing extensive necrosis and inflammation (A, H&E, 20x), tumor cells in sheets (B, H&E, 20x), cells with vacuolated cytoplasm and intracytoplasmic RBCs (C, H&E, 40x), and perineural invasion (D, H&E, 40x) H&E: hematoxylin and eosin, RBCs: red blood cells

Sections from the biopsy specimen showed extensive areas of necrosis and focal viable tumor cells arranged in sheets, cords, and nests with epithelioid and spindle cell morphology (Figure 2). Perineural invasion was noted. Individual cells showed an enlarged, round-to-oval nucleus and scant vacuolated cytoplasm. Cells with intracytoplasmic lumen were seen, some of which contained RBCs.

**Figure 3:**
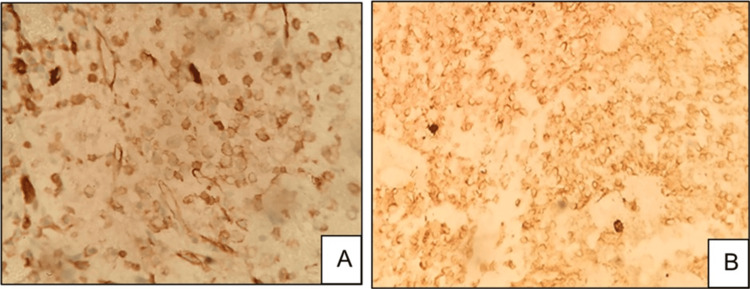
Tumor cells showing CD31 positivity (A, 40x) and CK positivity (B, 40x)

Immunohistochemistry showed positivity for cytokeratin and CD31 (Figure 3).

**Figure 4:**
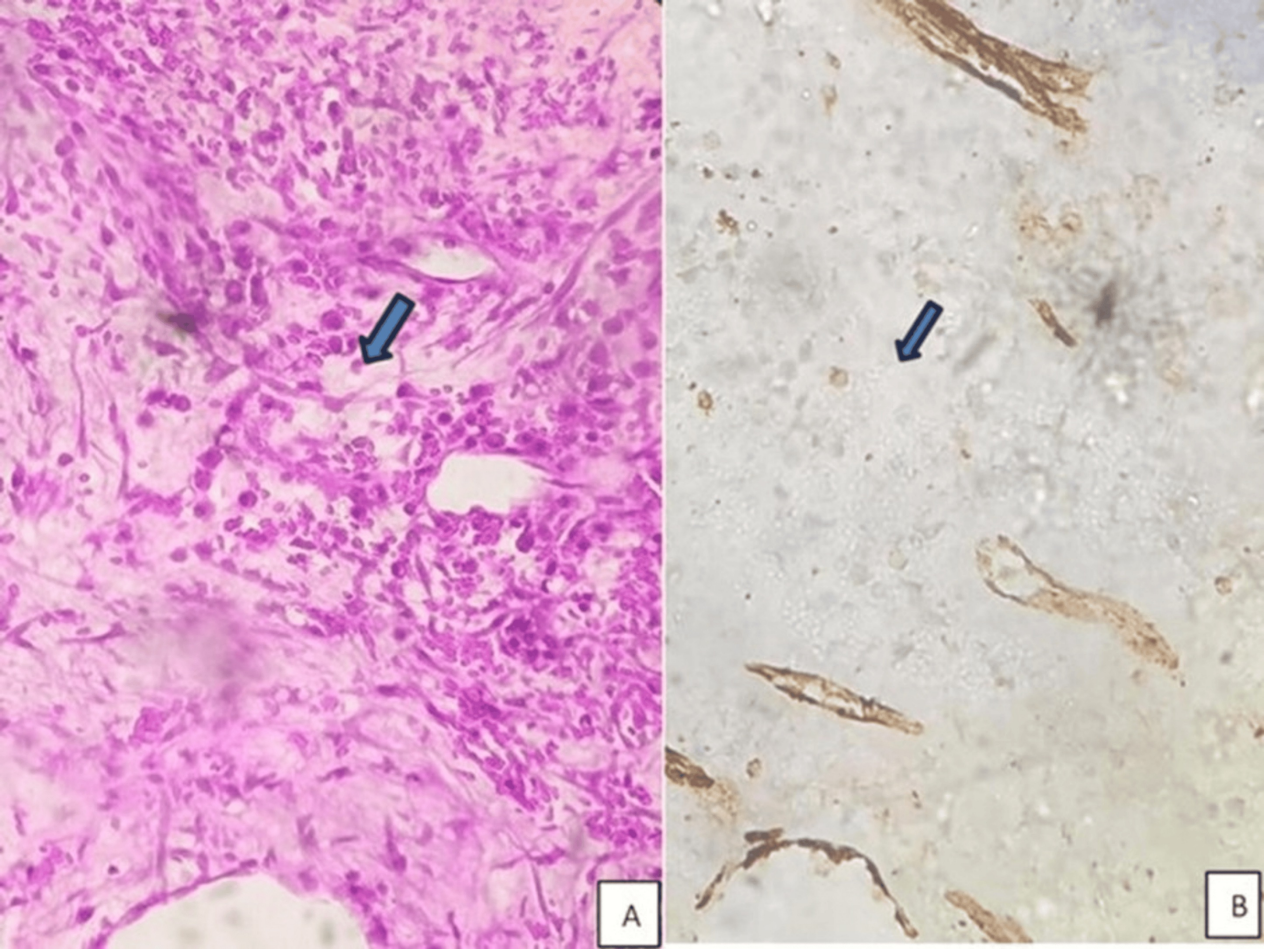
(A) Histiocyte collections in the initial biopsy specimen (H&E, 40x) and (B) CD31 not highlighting the histiocytes (CD31, 40x). Histiocytes indicated by blue arrows H&E: hematoxylin and eosin

In addition to endothelium, CD31 can also be positive in histiocytes. Care was taken to avoid misinterpreting such positivity as positive staining of tumor cells (Figure 4).

**Figure 5:**
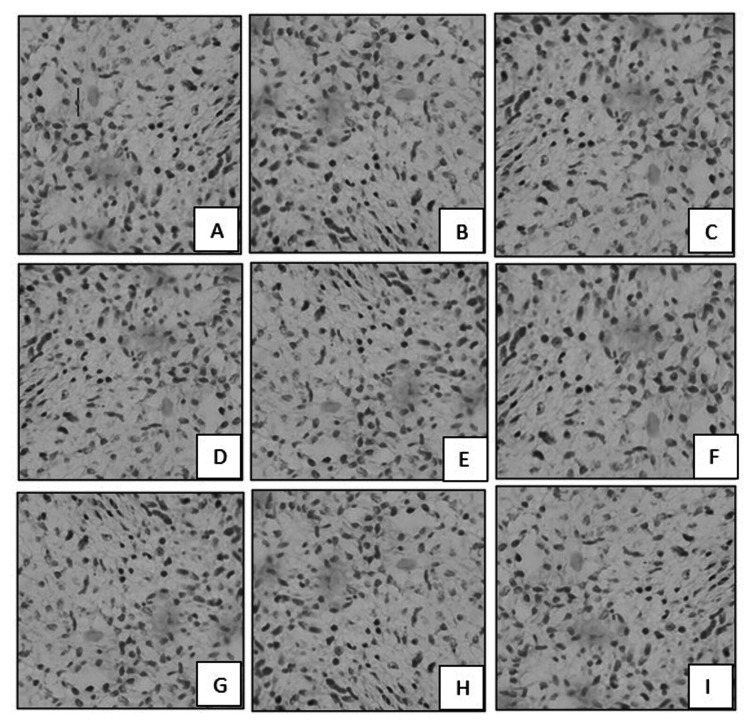
Tumor cells were negative for Myf4 (A, 40x), WT1 (B, 40x), synaptophysin (C, 40x), LCA (D, 40x), desmin (E, 40x), CD99 (F, 40x), CD34 (G, 40x), SMA (H, 40x), and S100 (I, 40x)

Tumor cells were also negative for Myf4, synaptophysin, WT1, LCA, S100, CD34, SMA, CD99, and desmin (Figure 5).

**Figure 6:**
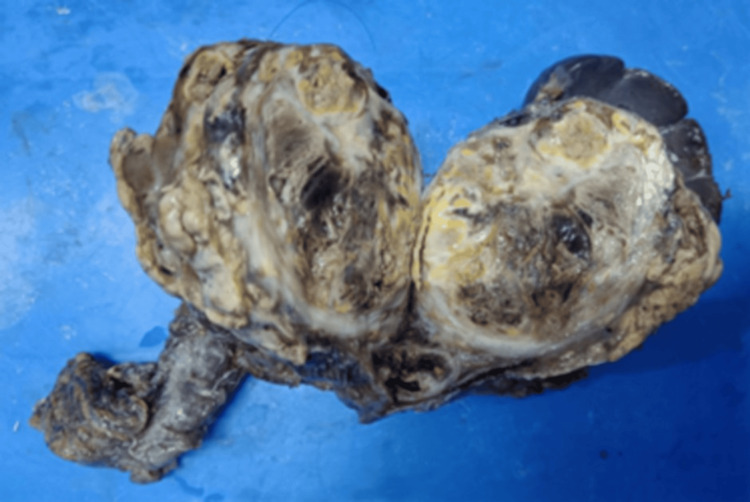
Gross examination showing a fairly circumscribed tumor with variegated appearance and central necrosis, infiltrating part of the bowel wall, and abutting the spleen but not infiltrating it grossly

Based on the morphology and IHC, epithelioid hemangioendothelioma (EHE) was considered as a possibility. Following the initial biopsy, the tumor was completely excised along with the spleen and part of the colon, with restoration of colonic continuity by end-to-end anastomosis (Figure 6). Grossly, the tumour was seen infiltrating the adjacent bowel wall, abutting the spleen, and adherent to part of the pancreas on the medial side.

**Figure 7:**
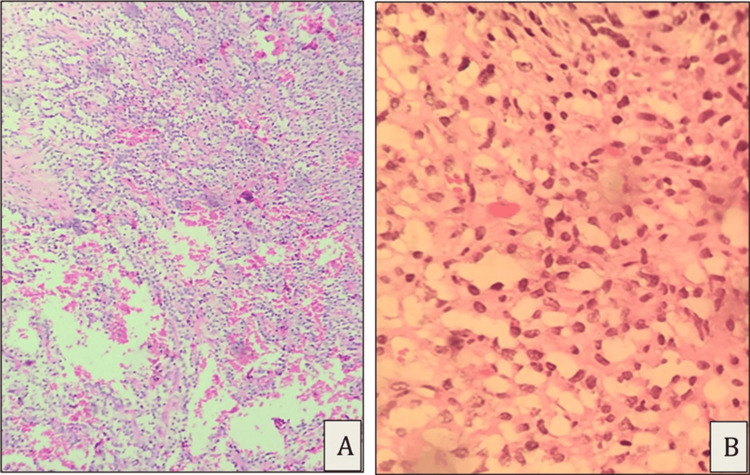
Excision specimen showed pseudopapillary arrangement of tumor cells focally (A, H&E, 20x) and tumor cells with clear cytoplasm and round hyperchromatic nuclei (B, H&E, 40x) H&E: hematoxylin and eosin

The sections showed large areas of necrosis with the viable tumor arranged in sheets and cords (Figure 7). A characteristic pseudopapillary pattern was noted, albeit focal. Hemosiderin deposition, sheets of foamy macrophages, calcification, and scattered cholesterol clefts, with occasional scattered osteoclast-like giant cells, were seen. There was no infiltration into the spleen.

**Figure 8:**
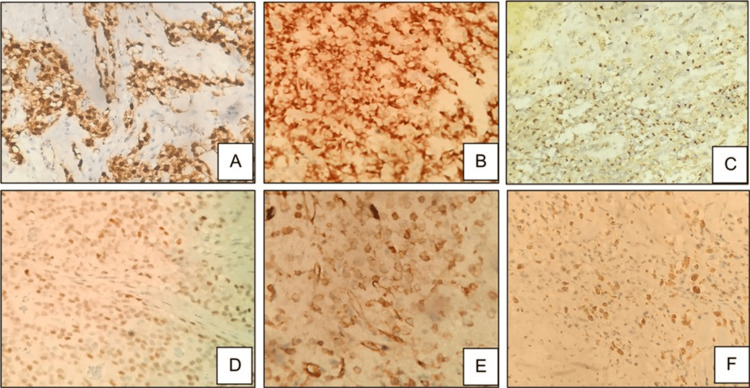
Tumor cells showing beta-catenin positivity (A, 40x), CK positivity (B, 40x), CD99 positivity (C, 40x), PR positivity (D, 40x), CD31 positivity (E, 40x), and CD56 positivity (F, 40x)

Based on morphological suspicion of SPN, targeted immunomarkers were performed, and the tumor cells were positive for CK, beta-catenin (nuclear), CD56, CD99 (dot-like), progesterone receptor (PR, focal), and CD31 (focal) (Figure 8). They were negative for ERG, synaptophysin, chromogranin, E-cadherin, WT1, CD34, and desmin. Ki-67 index was 1-2%. Based on these findings, a diagnosis of SPN was made.

**Figure 9:**
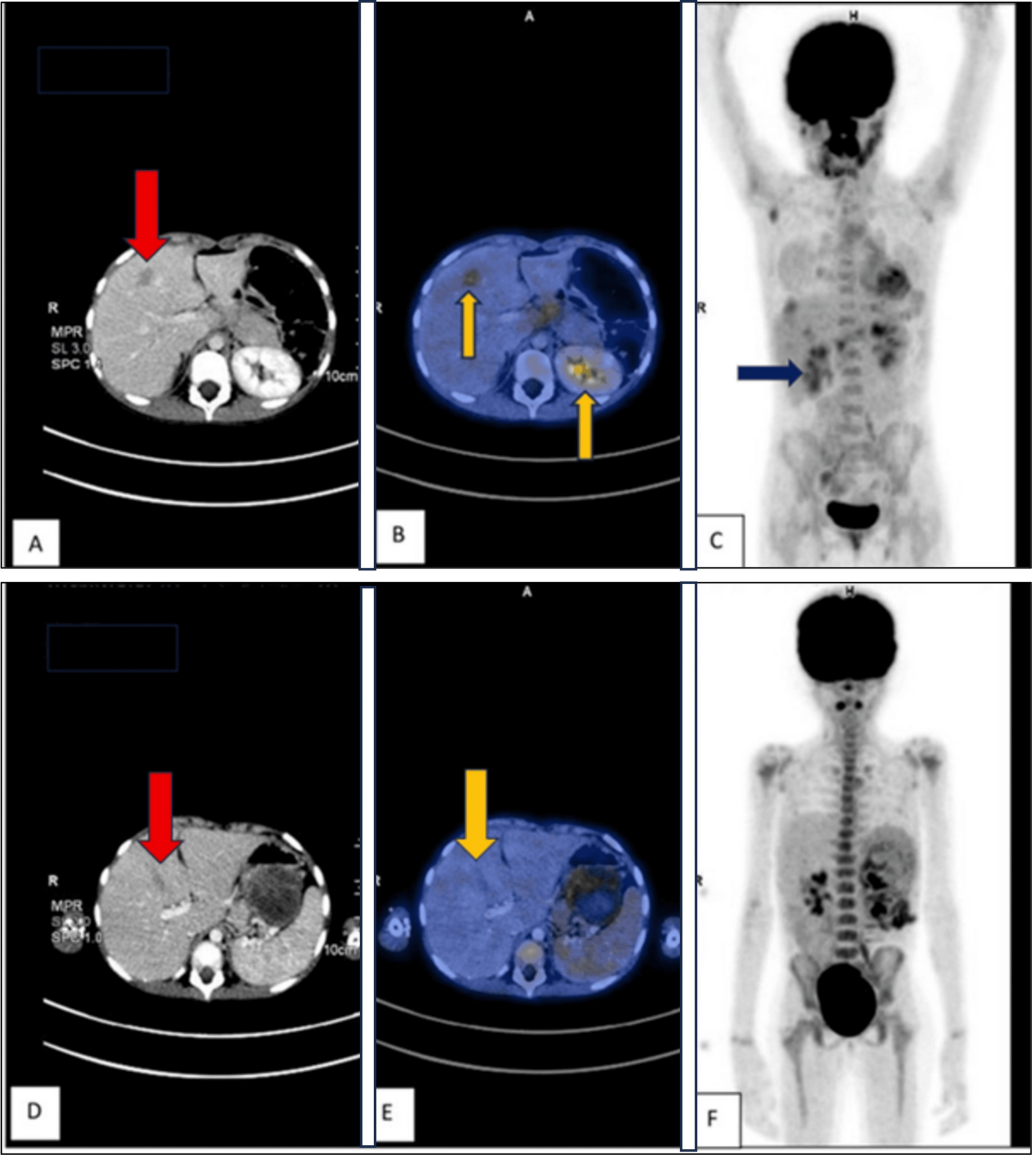
Two-year follow-up: (A) CT image showing a liver lesion with altered density (red arrow). (B) Fused PET-CT image highlighting areas of increased metabolic activity in the liver (yellow arrows), suggestive of active disease. (C) Whole-body PET image showing increased uptake in the liver region corresponding to (B) (blue arrow), indicating active disease. One-year follow-up: (D) CT image showing a smaller, less pronounced liver lesion (red arrow). (E) Fused PET-CT image demonstrating reduced metabolic activity compared with (B), suggesting less active disease. (F) Whole-body PET image showing limited or no significant metabolic activity in the liver or elsewhere, indicating a less active or earlier stage of disease PET: positron emission tomography, CT: computed tomography

On follow-up after one year, the patient presented with complaints of intermittent abdominal pain. A CT of the abdomen with contrast showed a well-defined hypoenhancing lesion in segment VII of the liver, along with multiple subcentimetric aortocaval, paraaortic, ileocolic, and portocaval lymph nodes. A CT of the abdomen performed at the two-year follow-up showed an increase in the size and number of liver lesions compared with the prior scan (Figure 9).

In view of the progression of the disease with metastatic spread not amenable to radiation therapy or surgery, the patient has been started on systemic chemotherapy. She is currently on gemcitabine and nab-paclitaxel.

## Discussion

SPN is widely regarded as a low-grade malignant pancreatic tumor with a distinctive clinicopathological and molecular profile. While most large series describe excellent long-term outcomes following complete surgical excision, emerging pediatric data suggest that SPN in children may exhibit a broader biological spectrum than previously appreciated, including locally aggressive behavior and, rarely, distant metastasis [1,6,7]. The present case contributes to this evolving understanding by highlighting both an unusual immunophenotypic feature and an aggressive clinical course in a very young patient.

Pediatric SPN remains a diagnostic challenge due to its rarity and frequent deviation from the classic radiological appearance seen in adults. Several pediatric series have reported that SPNs in children are more likely to be misinterpreted as mesenteric, retroperitoneal, or peritoneal tumors on imaging, particularly when they lack well-formed cystic components or demonstrate extensive necrosis [2,4,5]. In our case, preoperative imaging strongly favored a mesenteric or peritoneal sarcoma, consistent with prior reports emphasizing the limited specificity of radiological findings in pediatric SPN [3,6]. This reinforces the importance of maintaining SPN in the differential diagnosis of large upper abdominal masses in children, even when pancreatic origin is not readily apparent.

Histopathological diagnosis of SPN on limited biopsy material is well known to be difficult, especially in the presence of extensive necrosis and degenerative change [12]. The characteristic pseudopapillary architecture, considered a diagnostic hallmark, may be focal or entirely absent in small samples. In such circumstances, tumor cells may display epithelioid or spindle morphology, cytoplasmic vacuolization, and intracytoplasmic lumina, features that overlap with those of a variety of neoplasms, including vascular tumors, neuroendocrine neoplasms, and acinar cell carcinoma [9,12]. The initial biopsy in our case exemplifies this problem, as extensive necrosis and epithelioid morphology obscured the underlying diagnosis.

Immunohistochemistry plays a pivotal role in resolving such diagnostic dilemmas. The canonical immunophenotype of SPN-nuclear beta-catenin positivity due to CTNNB1 mutations, CD56 positivity, dot-like CD99 expression, progesterone receptor positivity, and loss of EE-cadherin has been well established across adult and pediatric cohorts [9-11]. These features were clearly demonstrated in the excision specimen in our case, confirming the diagnosis. However, focal CD31 positivity in tumor cells is a highly unusual finding that has not been well characterized in the SPN literature.

CD31 is a sensitive endothelial marker, and its expression is typically associated with vascular neoplasms, including EHE and angiosarcoma. Aberrant CD31 expression in epithelial tumors is rare but has been described sporadically in non-endothelial malignancies and histiocytic proliferations [13]. In the present case, CD31 positivity on the initial biopsy, combined with epithelioid morphology and intracytoplasmic lumina containing RBCs, raised a strong suspicion of EHE. This represents a significant diagnostic pitfall, particularly in limited-biopsy specimens in which architectural clues are lacking.

The distinction between SPN and EHE is not merely academic, as these entities differ substantially in biological behavior, prognosis, and management. EHE is an intermediate-grade malignant vascular neoplasm with a documented risk of local recurrence and metastasis, whereas SPN generally follows an indolent course with excellent outcomes after complete excision [7,8]. The use of additional vascular markers, particularly ERG and CD34, is therefore critical when CD31 positivity is encountered. In our case, ERG negativity effectively excluded a vascular lineage, underscoring the importance of a comprehensive and thoughtfully selected immunohistochemical panel.

From a prognostic standpoint, the development of metastatic disease in our patient is noteworthy. Although SPN is traditionally considered a tumor with low malignant potential, metastatic disease has been reported in approximately 5-15% of cases, most commonly involving the liver and peritoneum [6,7]. Pediatric cases with metastatic behavior appear to be particularly uncommon but are increasingly recognized, challenging the long-held perception of SPN as uniformly benign in children [7]. The low Ki-67 index in our case highlights the imperfect correlation between proliferative activity and clinical behavior, a phenomenon reported by others. It suggests that additional molecular or microenvironmental factors may influence tumor progression [12].

Recent advances in molecular pathology have begun to elucidate novel diagnostic and prognostic markers in SPN, including ABCD1 and emerging omics-based signatures, which may help refine risk stratification in the future [12,13]. However, these techniques are not yet routinely available in most diagnostic settings, reinforcing the ongoing importance of meticulous morphological assessment and judicious immunohistochemical interpretation.

## Conclusions

This case of SPN underscores the importance of heightened vigilance in recognizing unusual clinical presentations and the significance of interdisciplinary collaboration between radiologists, pathologists, surgeons, and oncologists. In addition to the classically described immunoprofile, our case displayed positivity for CD31, which has not been previously reported and is a potential diagnostic pitfall. Further studies are required to assess the frequency of CD31 positivity in SPN.
